# Ultrasound-Assisted Natural Deep Eutectic Solvent Extraction of Millet Polyphenols: Alleviating Effects on Oxidative Stress in *Caenorhabditis elegans* and Omics Analysis

**DOI:** 10.3390/foods15213958

**Published:** 2026-11-04

**Authors:** Junling Zhu, Jinghong Wang, Huimin Ma, Yakun Li, Yajing Xue, Xinyue Liu, Min Pan

**Affiliations:** 1 College of Food Science and Engineering, Shanxi Agricultural University, Taigu 030801, China; 202430727@stu.sxau.edu.cn (J.W.); 202430710@stu.sxau.edu.cn (H.M.); 202430703@stu.sxau.edu.cn (Y.L.); pheobe78@163.com (Y.X.); liuxinyue13@sxau.edu.cn (X.L.); panmin@sxau.edu.cn (M.P.); 2 Shanxi Province Key Laboratory Cultivation Base of Minor Crops Nutrition and Healthy Food Development, Taigu 030801, China

**Keywords:** millet polyphenols, *Caenorhabditis elegans*, oxidative stress, transcriptomics, metabolomics

## Abstract

Millet polyphenols are rich in nutrients and possess antioxidant, antibacterial, and hypoglycemic activities, but their in vivo mechanism remains unclear. In this study, a ternary natural deep eutectic solvent (NADES) was used as a green alternative to traditional solvents, and ultrasound-assisted extraction was applied to optimize purified millet polyphenol extract. *Caenorhabditis elegans* was used as an in vivo model to evaluate antioxidant activity. Growth and development, exercise capacity, lifespan, and antioxidant enzyme activities were measured, combined with transcriptome RNA-seq, RT-qPCR, and untargeted metabolomics to explore potential regulatory mechanisms. Results showed that purified millet polyphenol extract improved growth, development, and exercise capacity, and prolonged lifespan in stressed nematodes. They increased superoxide dismutase (SOD) and catalase (CAT) activities and decreased malondialdehyde (MDA) levels. The transcriptomic and metabolomic results suggest that the antioxidant effects of the purified foxtail millet polyphenol extract may be associated with changes in the Wnt, PI3K-Akt, and P53/P21 signaling pathways. At the same time, the remodeling of metabolite levels (such as glycerophospholipid metabolism and purine metabolism) also suggests that it may exert protective effects through a multi-pathway network. However, whether these pathways are activated and causally involved in the protective effects still requires functional experimental validation.

## 1. Introduction

The occurrence of many diseases is related to the oxygen free radicals in the human body, which can attack the mitochondria in nerve cells and damage the cells, thus causing neurological diseases [1]. Hydroxyl radicals have higher reactivity than other free radicals and are prone to react with other molecules, causing damage to DNA molecules [2]. The phenolic hydroxyl group in polyphenols can break the combination of hydrogen atoms and free radicals, thereby scavenging free radicals. A large number of studies have elucidated the antioxidant potential of natural polyphenol extracts from edible plants, such as grape seed polyphenols [3], green tea polyphenols [4] and pomegranate polyphenols [5].

Millet is one of the most important cereals with the longest planting history in China, which is rich in dietary fiber, protein, starch, vitamins, etc. [6]. Millet is rich in free and bound phenolic acids, such as hydroxybenzoic acid, hydroxycinnamic acid, etc., and also contains flavonoids, flavanols and isoflavones. In addition, some millet also contains a small amount of tannins [7]. Polyphenol compounds have health-promoting properties and play a vital role in preventing or reducing the occurrence of degenerative diseases, including osteoporosis, cancer, diabetes, neurological and cardiovascular diseases [8]. Therefore, it is necessary to deeply excavate and utilize millet resources. As a new type of natural active substance, millet polyphenol has become a hot spot in the field of phytochemistry, and also shows good development potential in the field of medicine.

*Caenorhabditis elegans* (*C. elegans*) has a short growth cycle and survives at 20 °C for 2–3 weeks [9]. It is easy to be cultured in the laboratory, greatly shortens the experimental period, and facilitates experimental design and repetitive operation. The nematode body is translucent, and the structure is simple. Its survival state, appearance characteristics and internal structure can be seen under the microscope. Among all the model organisms, *C. elegans* is the first species to complete the whole genome sequencing. Its complete genome sequence is available, and about 50% of the human protein coding sequences have recognizable homologous genes in nematodes [10]. Many studies have successfully identified various anti-aging compounds and elucidated their mechanisms of action in *C. elegans*. Studies have shown that [11], rutin can activate heat shock proteins in nematodes, promote the expression of daf-16, enhance its anti-oxidative stress ability and prolong survival time. Studies have found that oral administration of dark tea can significantly reduce the level of reactive oxygen species and show a longer lifespan in H_2_O_2_-induced oxidative stress in *C. elegans* [12]. Flavonoid extracts from citrus peel and Pongamia pinnata can alleviate the oxidative stress injury of *C. elegans*, and have a good application prospect in neurodegenerative diseases [13,14]. In order to fill the gap in knowledge about the antioxidant activity of millet polyphenols, a comprehensive study was conducted on its effect on *C. elegans*.

In this study, millet was used as raw material, and the experimental methods established by the research group were used to prepare polyphenols, separate active components and determine their basic physical and chemical properties. The oxidative stress model of *C. elegans* was established, and the effects of purified millet polyphenol extract on physiological indexes, transcriptome and metabolome of nematodes were systematically analyzed. The results of this study will provide an important reference for the research and development of deep processing technology and product development and innovation of purified millet polyphenol extract and grain polyphenols. This study will also provide an important reference for the search and product development of antioxidant natural active ingredients.

## 2. Materials and Methods

### 2.1. Chemicals and Strains

*C. elegans* (N2) and *Escherichia coli* (*E. coli* OP50) were purchased from Fujian Shangyuan Biological Science and Technology Co., Ltd. (Fuzhou, China).

### 2.2. Preparation of Millet Polyphenol Extract

**Figure 1:**
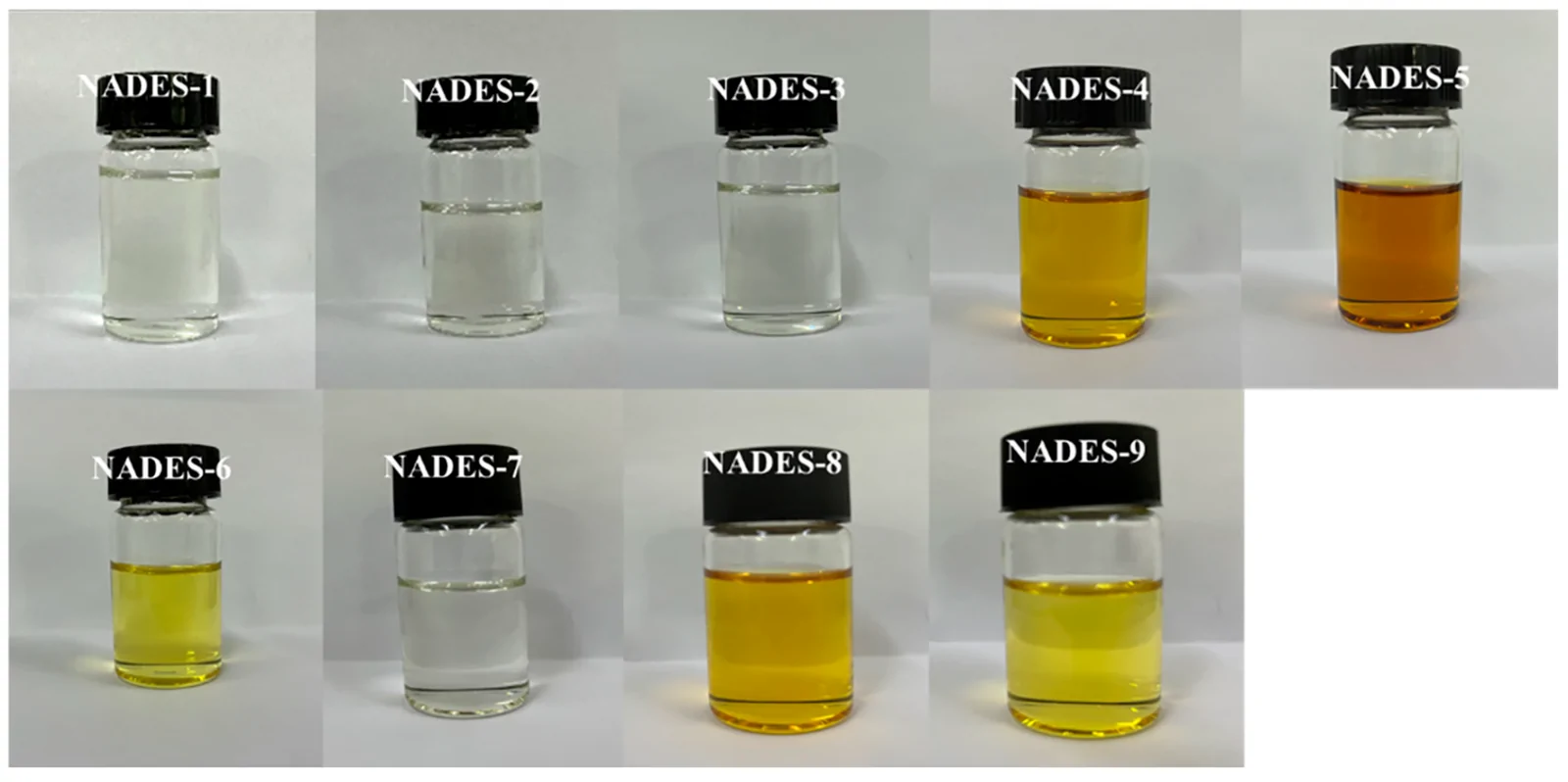
9 kinds of NADES preparation diagrams.

To efficiently extract foxtail millet polyphenols, this study first screened nine ternary natural deep eutectic solvents (NADES-1 to NADES-9) (Table 1). The visual appearances of these prepared NADES formulations are shown in Figure 1. Because differences in polarity, pH, and hydrogen-bond networks among NADES systems can significantly affect the extraction efficiency of bioactive compounds [15,16], polyphenol extraction yield was used as the evaluation indicator. The results showed that NADES-2 (glycerol/malic acid/glucose, molar ratio 1:1:1) had the highest extraction efficiency and was therefore selected as the extraction solvent for subsequent experiments. On this basis, the extraction parameters were further optimized, including the molar ratio (1:2:1, 2:1:1, 3:2:1, 4:2:1, 5:2:1), water content (20%, 30%, 40%, 50%, 60%, 70%), liquid–solid ratio (20, 30, 40, 50, 60 mL/g), ultrasonic time (20, 30, 40, 50, 60 min), and extraction temperature (30, 40, 50, 60, 70 °C). The optimum process was as follows: 0.1 g defatted millet flour was weighed and added with 2 mL NADES-2 (glycerol/malic acid/glucose molar ratio of 3:2:1) with 30% water content. After mixing, it was placed in an ultrasonic cleaner and ultrasonically extracted at 63 °C for 39 min. After extraction, the supernatant was centrifuged at 8000 rpm for 15 min. Under these conditions, the extraction amount of millet polyphenols could reach 8.23 mg/g.

The crude extract of millet polyphenols was wetly loaded on the column, and the impurities were removed by distilled water, so that the polyphenols were adsorbed on the macroporous resin, and 70% ethanol was desorbed. The purified polyphenols were collected, evaporated and concentrated, filtered through a 0.45 μm filter membrane, and freeze-dried into powder. After purification, the polyphenol purity was 73.8% ± 0.62%. When the concentration of polyphenols was 14 mg/mL, the scavenging rates of DPPH and ABTS· free radicals were 52.25% and 64.48%, respectively, which provided data support for the subsequent application of millet polyphenols. No separate NADES blank control or individual NADES component controls were applied during the extraction and purification steps.

In the early stage of our research group, chemical identification, infrared spectroscopy, ultraviolet-visible spectroscopy and liquid chromatography-mass spectrometry were used to analyze the components of purified millet polyphenols. It was speculated that it contained flavonoids, mainly hydroxyl caffeic acid, cyanidin-3-O-galactoside chloride and p-coumaroyl malic acid [17].

### 2.3. Cultivation of Caenorhabditis elegans

The culture of *C. elegans* fed on uracil-deficient *Escherichia coli* OP50, which grew slowly and only formed a thin layer on the surface of NGM, which did not interfere with the normal development of nematodes. Before culture, the frozen OP50 was activated, monoclonal cultured and coated on NGM plate for later use. The nematode cryopreservation tube was thawed in a water bath at 37 °C, centrifuged to remove the supernatant, resuspended in buffer, transferred to an NGM plate containing OP50, and activated at 20 °C. When synchronization was required, the oviposition adults were collected and treated with lysis buffer to release eggs. After washing, the eggs were transferred into fresh OP50 plates and cultured at 20 ° C for 48 h to obtain L4 larvae. The experiment was divided into normal group, model group and polyphenol group (100, 200 and 400 μg/mL).

### 2.4. Determination of Physiological Characteristics of Caenorhabditis elegans

In order to evaluate the physiological response of nematodes to purified millet polyphenol extract, exercise capacity measurement and longevity experiments were carried out. Ten nematodes cultured for 5 days were randomly selected from the normal group, model group, and polyphenol groups at various concentrations and placed on empty NGM plates without food. Images were taken under a stereomicroscope, and body length and body width were measured using ImageJ software (version 1.54r, U.S. National Institutes of Health, Bethesda, MD, USA). Body bends and head thrashes within 1 min were also recorded to evaluate locomotor ability. Body length was defined as the straight-line distance from the anterior end of the head to the posterior end of the tail, and body width was defined as the perpendicular distance at the widest part of the body. To minimize observer-related variation, each image was measured three times and the average value was used.

The lifespan assay was performed on NGM plates containing OP50 and 150 μM 5-fluorouracil (to inhibit reproduction). Nematodes in the control group were cultured normally. The model group was treated with 1000 μM H_2_O_2_ to induce oxidative stress. The polyphenol groups received the same concentration of H_2_O_2_ supplemented with the corresponding concentrations of purified millet polyphenol extract. Fresh plates with the respective treatments were replaced every 48 h. Nematode survival was monitored daily starting from the day of transfer, and the number of surviving worms and survival days were recorded. Individuals that died from wall climbing or accidental death were excluded from the statistics. Three parallel plates were set up for each group, with approximately 30 nematodes per plate.

### 2.5. Determination of Oxidative Stress-Related Enzyme/Non-Enzyme Levels in Nematodes

To evaluate the ability of the purified millet polyphenol extract to enhance the oxidative stress tolerance of nematodes, synchronized L4-stage nematodes were treated with different doses of the extract, and an untreated control group was set up. After 5 days of polyphenol treatment, the nematodes were transferred to medium containing 1000 μM H_2_O_2_ for 48 h of oxidative stress exposure. Subsequently, the nematodes were collected into EP tubes and allowed to settle naturally. They were washed 3–5 times with M9 buffer, and the supernatant was discarded after centrifugation. Pre-cooled normal saline was added to the precipitate, and tissue homogenates were obtained by tissue grinding, followed by centrifugation to collect the supernatant. First, the total protein concentration of the supernatant was measured using a BCA kit. Then, the absorbance at specific wavelengths was measured according to the instructions of the SOD, CAT, and MDA kits, and each index was calculated using the respective formulas.

### 2.6. Oxidative Stress Tolerance Test

To verify the antioxidant stress effect of the purified foxtail millet polyphenol extract on *C. elegans*, synchronized nematodes were pretreated with polyphenols for 5 days and then transferred to acute stress medium containing 0.1% H_2_O_2_, where survival was observed and recorded hourly. For these two tests, each treatment group used 5 replicate plates, each plate containing 30 worms, to ensure statistical significance.

### 2.7. Establishment of Oxidative Stress Model in Nematodes

This step was only performed to determine the optimal H_2_O_2_ concentration for model establishment in preliminary experiments; it was not part of the subsequent polyphenol stress resistance test. The synchronized L4 stage nematodes were exposed to NGM medium containing different concentrations of H_2_O_2_ for 48 h. The adult was taken out and washed three times with buffer solution, and the MDA level was determined by crushing. If the MDA content increased significantly, the model was established successfully.

### 2.8. Transcriptome of Caenorhabditis elegans

In order to explore the mechanism of polyphenols on the health status of nematodes and analyze its effect on the overall transcription level and pathway, L4-stage synchronized *C. elegans* were randomly divided into three groups: the Control group, the Model group, and the polyphenol-treated group (MD). The Control and Model groups were cultured on standard NGM plates, while the MD group was continuously cultured on NGM plates containing the optimal concentration of purified millet polyphenol extract for 5 days. After this 5-day culture period, both the Model and MD groups were simultaneously transferred to NGM medium containing 1000 μM H_2_O_2_ for 48 h of oxidative stress exposure before sample collection. The Control group was collected at the corresponding time point as a blank control, rinsed with buffer solution for 3–5 times, centrifuged, frozen in liquid nitrogen and transferred to −80 °C for cryopreservation. Shanghai Meiji Biotechnology Co., Ltd. (Shanghai, China) was entrusted to perform transcriptome sequencing on *Caenorhabditis elegans*. The specific process was as follows: total RNA was extracted from the sample and subjected to quality inspection, followed by enrichment and fragmentation of mRNA, which was used as a template for reverse transcription to synthesize cDNA. After end repair, A tail and adaptor ligation, the library fragments were enriched by PCR, the library construction was completed, and then high-throughput sequencing was performed. The original sequencing data were filtered to obtain high-quality sequences, compared with the reference genome, the gene expression was calculated, and functional enrichment and expression pattern clustering analysis were performed. Finally, the antioxidant-related genes were selected and verified by real-time fluorescence quantitative PCR. According to the kit instructions, act-1 was used as the internal reference gene, and 2^−ΔΔCt^ value was used for quantitative analysis.

### 2.9. Non-Targeted Metabolomics of Caenorhabditis elegans

In order to explore the effect of polyphenols on the overall metabolic level of nematodes, a non-targeted metabolomics method based on UHPLC-Q Exactive HF-X was used for analysis. Each sample was weighed into a centrifuge tube, added with a grinding bead, and added with 400 μL of pre-cooled extract (methanol:water = 4:1, containing 0.02 mg/mL internal standard) to extract metabolites; all samples were mixed in equal volume to prepare quality control (QC) samples, and one QC sample was inserted for each analysis of 6 samples to test the repeatability of the whole process of the experiment. LC-MS analysis was carried out. The chromatographic conditions were as follows: HSS T3 column (100 mm × 2.1 mm, 1.8 μm), column temperature 40 °C, flow rate 0.40 mL/min, injection volume 3 μL; mobile phase A was 95% water, 5% acetonitrile and 0.1% formic acid, mobile phase B was 47.5% acetonitrile, 47.5% isopropanol, 5% water and 0.1% formic acid, gradient elution. The collected original data were subjected to peak identification, integration, retention time correction and peak alignment by software to form a data matrix. After eliminating outliers and standardizing them, principal component analysis (PCA) and orthogonal partial least squares discriminant analysis (OPLS-DA) were performed. The stability of the OPLS-DA model was evaluated by 7-fold cross-validation. Metabolites with variable importance in projection (VIP) > 1 and Benjamini–Hochberg (BH)-adjusted q < 0.05 were selected as differential metabolites. Because no authentic standards were used, these metabolites are reported as MSI Level 2 putative annotations.

### 2.10. Statistical Analysis

All statistical analyses were performed using GraphPad Prism 9. In order to determine the significant difference between the experimental groups, one-way ANOVA or two-way ANOVA was used to test the significance between the experimental groups. Note: Significance was calculated as *p* < 0.05, *n* = 3. Different letters indicated that the significance of the mean was different (*p* < 0.05). For life analysis, the log-rank (Kaplan–Meier) statistical test was used to calculate the average life expectancy and to evaluate the statistically significant differences between the groups. All data were expressed as mean ± SD to provide measurements of variability within each group.

## 3. Results

### 3.1. Effects of Polyphenols on Body Length and Body Width of Caenorhabditis elegans

**Figure 2:**
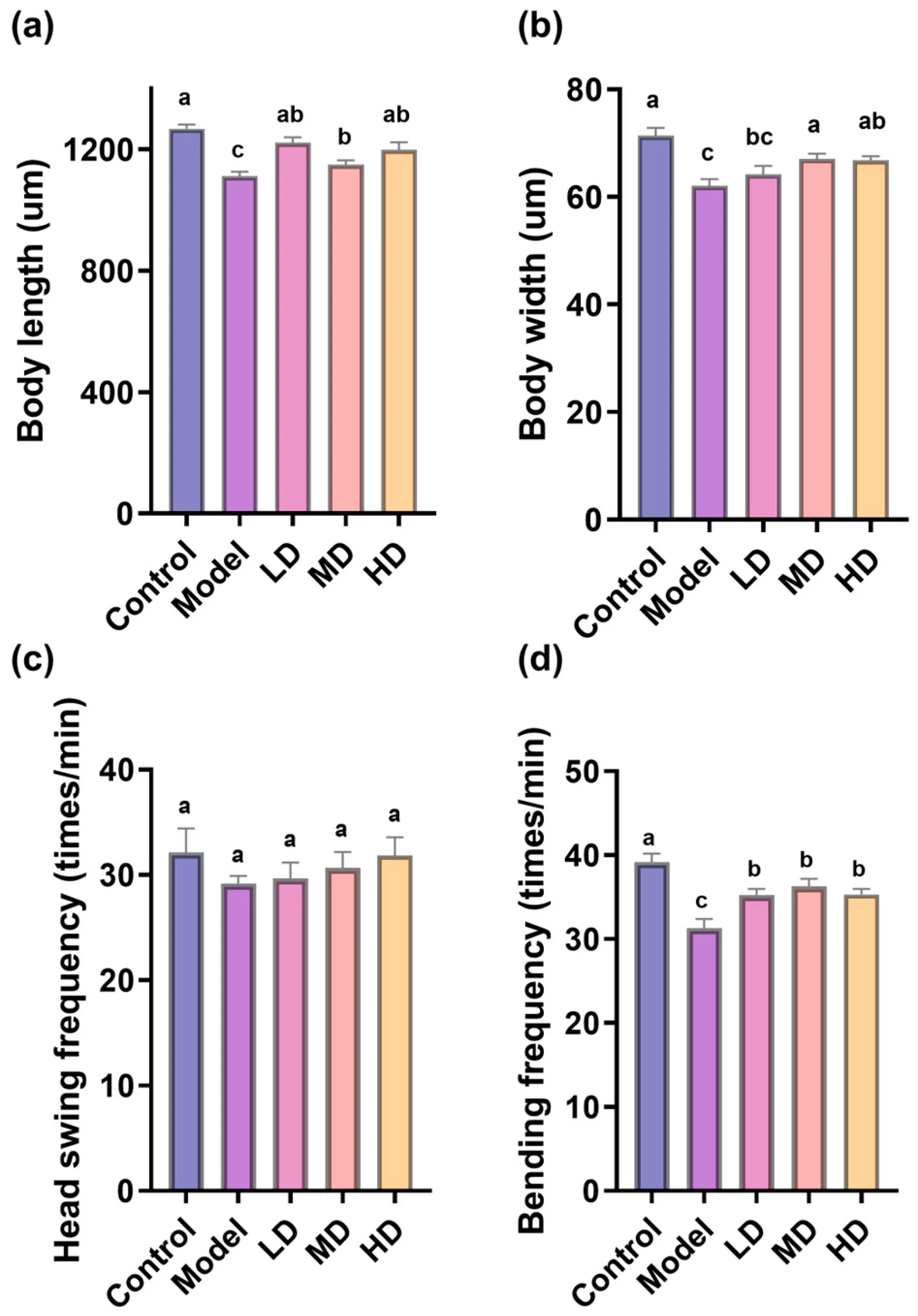
Effects of different concentrations of purified millet polyphenol extract on body length, body width, and locomotor behaviors of *Caenorhabditis elegans*. (**a**) Body length (μm); (**b**) body width (μm); (**c**) head swing frequency (times/min); (**d**) bending frequency (times/min). Data are presented as mean ± standard deviation. Different lowercase letters above the bars indicate statistically significant differences between groups (*p* < 0.05, using one-way ANOVA followed by post hoc tests). Groups sharing the same letter are not significantly different, whereas groups with completely different letters are significantly different.

The basic physiological indexes such as body length and body width of nematodes can reflect the growth status of nematodes to a certain extent due to factors such as food and growth environment [18]. Figure 2a,b is the effect of purified millet polyphenol extract on the body length and body width of nematodes. The results showed that the body length and body width of nematodes in the model group were significantly lower than those in the normal group, which were 1161.24 ± 62.09 and 61.78 ± 3.2, respectively, decreased by 9.12% and 12.25%. Compared with the model group, the body length and body width of nematodes were increased after polyphenol treatment. The body length and body width of nematodes in the LD group were increased by 7.34% and 3.02%, respectively. The body length and body width of nematodes in the MD group were increased by 4.76% and 9.3%, respectively. The body length and body width of nematodes in the HD group were increased by 7.0% and 8.81%, respectively, indicating that H_2_O_2_ had an inhibitory effect on the body length and body width of nematodes. It aggravates the oxidative stress level of nematodes, affects the integrity of cell structure and function, and further inhibits its growth and development [19], while polyphenols can alleviate its adverse effects on growth and development.

### 3.2. Effect of Polyphenols on the Movement Behavior of Caenorhabditis elegans

The main indicators for measuring the athletic ability of nematodes are head swing and body bending frequency, and athletic ability can reflect energy consumption [20]. When the nematode moves forward, the body mostly bends in a sinusoidal shape. The head swing frequency can reflect the aging of the nematode to a certain extent. The head swing is often related to feeding ability and muscle nerve ability. As shown in Figure 2c, no statistically significant differences in head thrashing frequency were observed among the groups (*p* > 0.05). Although the HD group showed a numerically slightly higher value than the model group (a 7.59% increase), this suggests that polyphenol treatment had a limited effect on head thrashing frequency in *C. elegans*. According to Figure 2d, there were significant differences in the body bending frequency of nematodes in each group. Compared with the normal group, the body bending frequency of nematodes in the model group decreased by 21.91%. Compared with the model group, the body bending frequency of nematodes in the LD group increased by 11.94%, the body bending frequency of nematodes in the MD group increased by 18.71%, and the body bending frequency of nematodes in the HD group increased by 12.9%. The preliminary results showed that purified millet polyphenol extract had a certain alleviating effect on the motor behavior defects of nematodes caused by H_2_O_2_ stimulation.

### 3.3. Effects of Polyphenols on Lifespan of Caenorhabditis elegans

**Figure 3:**
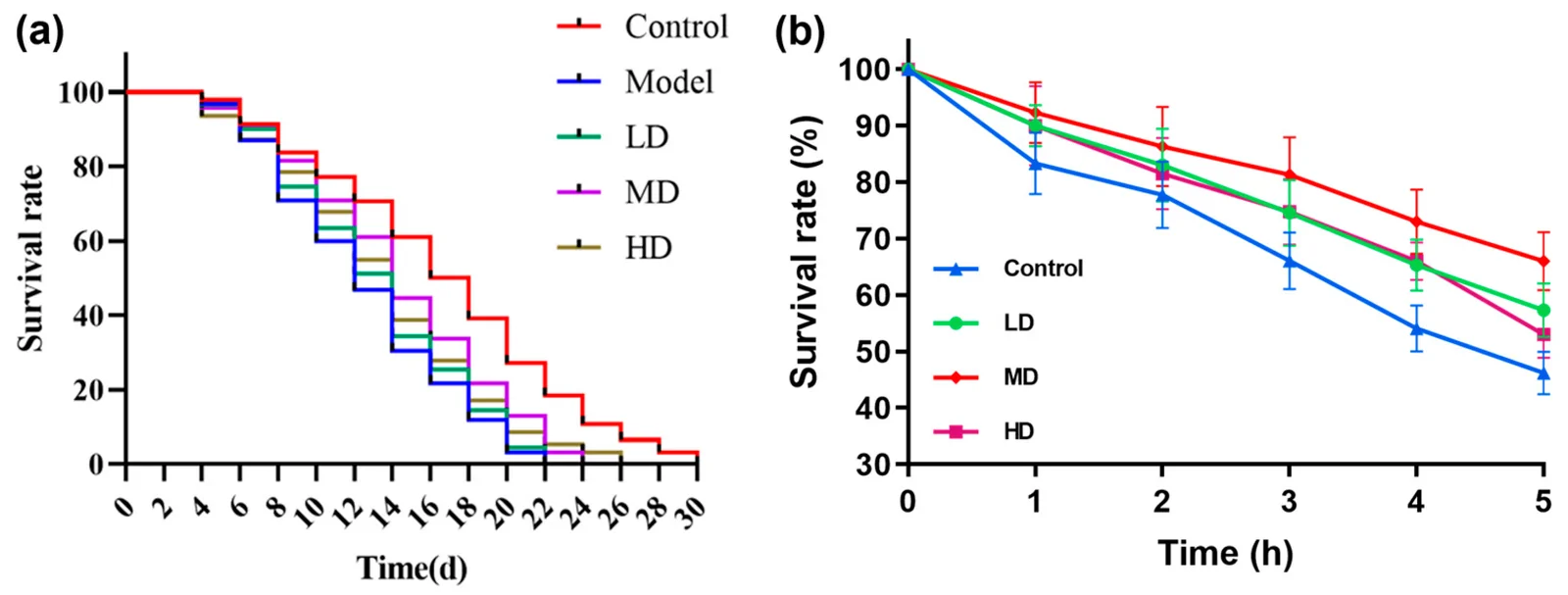
Effects of purified millet polyphenol extract on the survival rate of *Caenorhabditis elegans*. (**a**) Lifespan curves of nematodes under different concentration treatments (days); (**b**) survival curves of nematodes under stress conditions with different concentration treatments (hours). Figure (**a**) represents Kaplan–Meier survival curves, and intergroup differences were analyzed using the Log-rank test; data in Figure (**b**) are presented as mean ± standard deviation, and intergroup differences were analyzed using one-way ANOVA.

To investigate the effects of polyphenols on the lifespan of *C. elegans* under oxidative stress, survival assays were performed. As shown in Figure 3a, compared with the blank control group (mean lifespan of 17.54 days), the survival curve of the model group shifted to the left, and the mean lifespan was significantly shortened to 12.5 days (Log-rank test, *p* = 0.002), indicating that H_2_O_2_ exposure induced marked oxidative stress damage in the nematodes. Compared with the model group, the survival curves of the groups treated with different concentrations of purified foxtail millet polyphenol extract showed a certain rightward trend. Numerically, the MD group exhibited the largest lifespan extension, with the mean lifespan extended to 15.80 days (a 26.4% increase) and a maximum lifespan of 24 days. The HD group showed a similar but slightly lower performance, with a mean lifespan of 15.66 days (a 25.3% increase) and a maximum lifespan of 26 days. The mean lifespan of the LD group was 14.47 days (a 15.8% increase). Notably, the Log-rank trend test did not reveal a significant linear dose-dependent effect (*p* = 0.5914), suggesting that the lifespan-extending effect of the purified extract may not be a simple linear concentration dependence, but rather a non-monotonic dose response. Overall, these results suggest that intervention with the purified foxtail millet polyphenol extract may help alleviate H_2_O_2_-induced lifespan shortening, with the MD group exhibiting a relatively optimal intervention potential in terms of overall indicators; however, its exact dose–effect relationship requires further validation with larger sample sizes.

### 3.4. Analysis of Polyphenols on Anti-Oxidative Stress of Caenorhabditis elegans

In order to verify the anti-oxidative stress effect of purified millet polyphenol extract on *C. elegans*, after feeding *C. elegans* for 5 days, it was transferred to a medium supplemented with 0.1% H_2_O_2_ to observe the survival rate of *C. elegans* (Figure 3b). According to the survival curve of nematodes, compared with the blank group, the average lifespan of nematodes in the polyphenol treatment group was prolonged by 10.86%, 18.24% and 11.09%, respectively, indicating that polyphenols had an obvious ability to resist H_2_O_2_. Many studies have shown that the higher the survival rate of nematodes under oxidative stress, the longer their lifespan. It has been reported [21] that polyphenols such as flavonoids, flavonoids and coumarins can significantly improve functions such as oxidative stress, inflammation and cell senescence. In summary, polyphenols can resist the adverse environment of hydrogen peroxide by reducing the oxidative stress index of nematodes, thereby increasing the oxidative stress ability of nematodes and prolonging their survival time.

### 3.5. Effects of Polyphenols on H_2_O_2_-Induced Oxidative Stress-Related Enzymes in Caenorhabditis elegans

**Figure 4:**
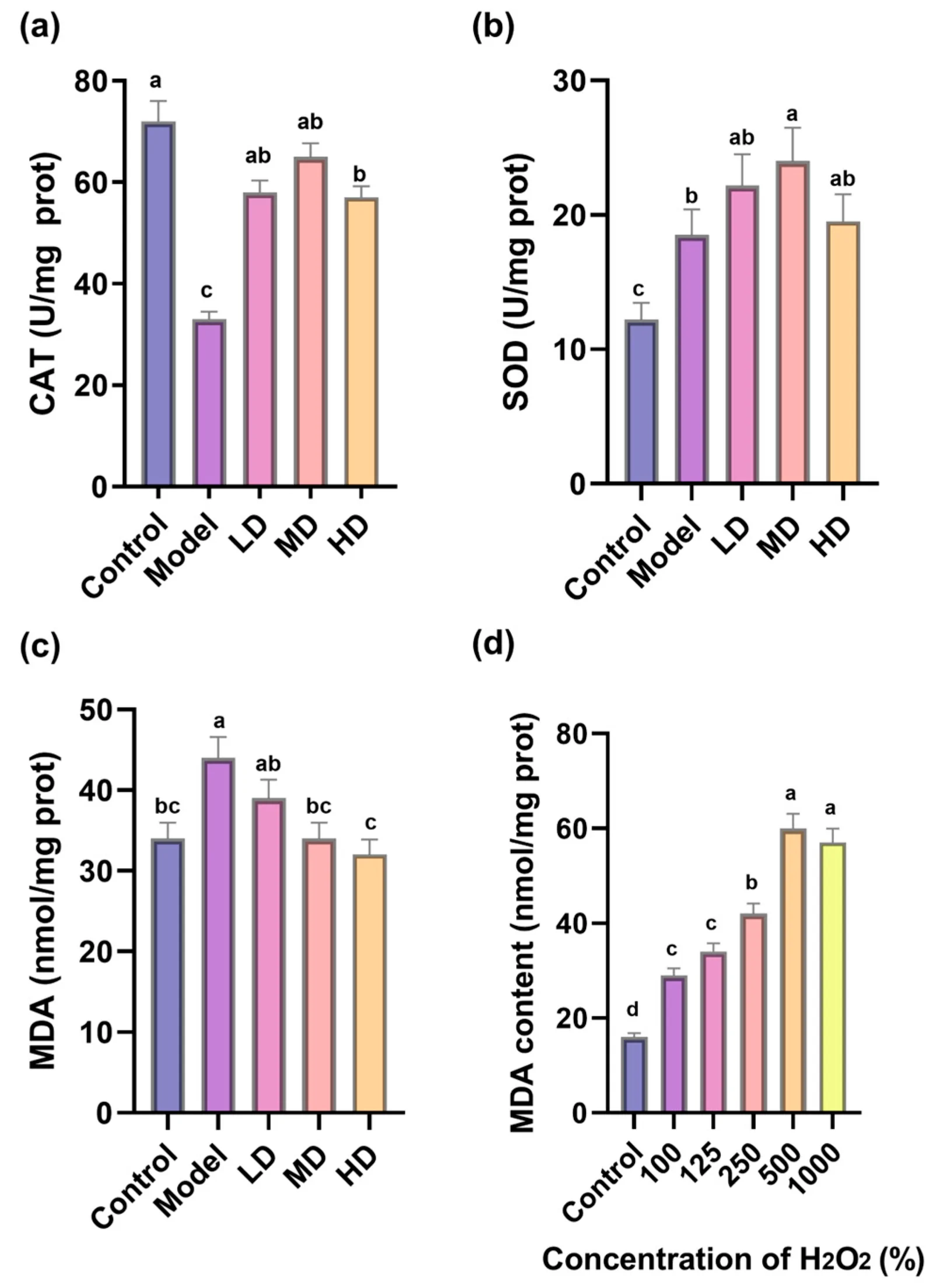
Effects of purified millet polyphenol extract on the antioxidant capacity of *Caenorhabditis elegans* and the level of H_2_O_2_-induced lipid peroxidation. (**a**) Catalase (CAT) activity; (**b**) superoxide dismutase (SOD) activity; (**c**) malondialdehyde (MDA) content; (**d**) MDA content under induction by different concentrations of H_2_O_2_. Data are presented as mean ± standard deviation. Different lowercase letters above the bars indicate statistically significant differences between groups (*p* < 0.05, using one-way ANOVA followed by post hoc tests). Groups sharing the same letter are not significantly different, whereas groups with completely different letters are significantly different.

Superoxide dismutase (SOD) and catalase (CAT) are important antioxidant enzymes in *C. elegans*, which can scavenge excessive free radicals and delay aging. They are important indicators for evaluating the aging degree of *C. elegans* [22,23]. Malondialdehyde (MDA) is one of the free products of polyunsaturated fatty acid peroxidation in cells. Its content can reflect the level of free radicals in the body and indirectly reflect the aging of cells, so it can be used as an important marker of aging [24]. After H_2_O_2_ treatment, the CAT activity of the model group was significantly lower than that of the normal group (Figure 4a). Compared with the model group, the activity of the polyphenol group increased by 70.90%, 92.69% and 68.78%, respectively. The SOD activity of the model group was 52.28% higher than that of the control group. The effects of polyphenols on SOD activity in *C. elegans* were increased by 19.92%, 31.15% and 5.24%, respectively, compared with the model group (Figure 4b). Studies have shown that ginger extract with anti-aging effect can significantly increase SOD activity in nematodes [25]. Therefore, the antioxidant effect of the purified foxtail millet polyphenol extract may be associated with a significant increase in SOD activity in *C. elegans*. In the model group, increased SOD activity accompanied by decreased CAT activity may reflect an imbalance in the antioxidant defense system. The elevation in SOD activity may represent a compensatory response to superoxide anions, whereas CAT, which is mainly responsible for H_2_O_2_ clearance, is more prone to oxidative inactivation under excessive H_2_O_2_ stress [26,27]. This differential response between SOD and CAT may exacerbate H_2_O_2_ accumulation, consistent with the elevated MDA levels observed in the model group. After polyphenol treatment, CAT activity was markedly restored, suggesting that polyphenols may help maintain the balance of the antioxidant system by alleviating oxidative damage to CAT. In addition, compared with the blank group, the MDA content in the model group was significantly increased, and the polyphenol treatment could significantly reduce the MDA content in the nematodes, which was reduced by 9.46%, 21.9% and 26.53%, respectively (Figure 4c), indicating that polyphenols can inhibit H_2_O_2_-induced lipid oxidative damage in nematodes.

### 3.6. Establishment of Oxidative Stress Model

Studies have shown that H_2_O_2_ can induce oxidative stress in cells or nematodes [28]. In this experiment, H_2_O_2_ was used to induce oxidative stress in nematodes and the content of MDA was determined. As shown in Figure 4d, with the increase in H_2_O_2_ concentration, the content of MDA in *C. elegans* increased significantly, and the concentration of H_2_O_2_ at 1000 μM had little effect on the survival of *C. elegans*. Therefore, H_2_O_2_ with a concentration of 1000 μM was selected to establish an oxidative stress model.

### 3.7. Effect of Millet Polyphenols on the Transcriptome of Caenorhabditis elegans

The intervention effects differed among the polyphenol treatment groups. The HD group was most prominent in improving locomotion and reducing lipid peroxidation, whereas the MD group performed best in oxidative stress resistance, SOD/CAT activity, and lifespan extension. Although changes in some enzyme activities were more pronounced in the LD group than in the MD and HD groups, its improvements in whole-organism phenotypes were relatively weak. After weighing all physiological and biochemical indicators, this study ultimately selected the MD group for subsequent transcriptomic and metabolomic analyses. Objectively, although omics analysis using a single MD concentration can provide reliable molecular evidence under this condition, it cannot fully characterize dose-dependent effects, and the related molecular features should not be directly extrapolated. This limitation still needs to be addressed in future studies through multi-concentration experiments.

**Figure 5:**
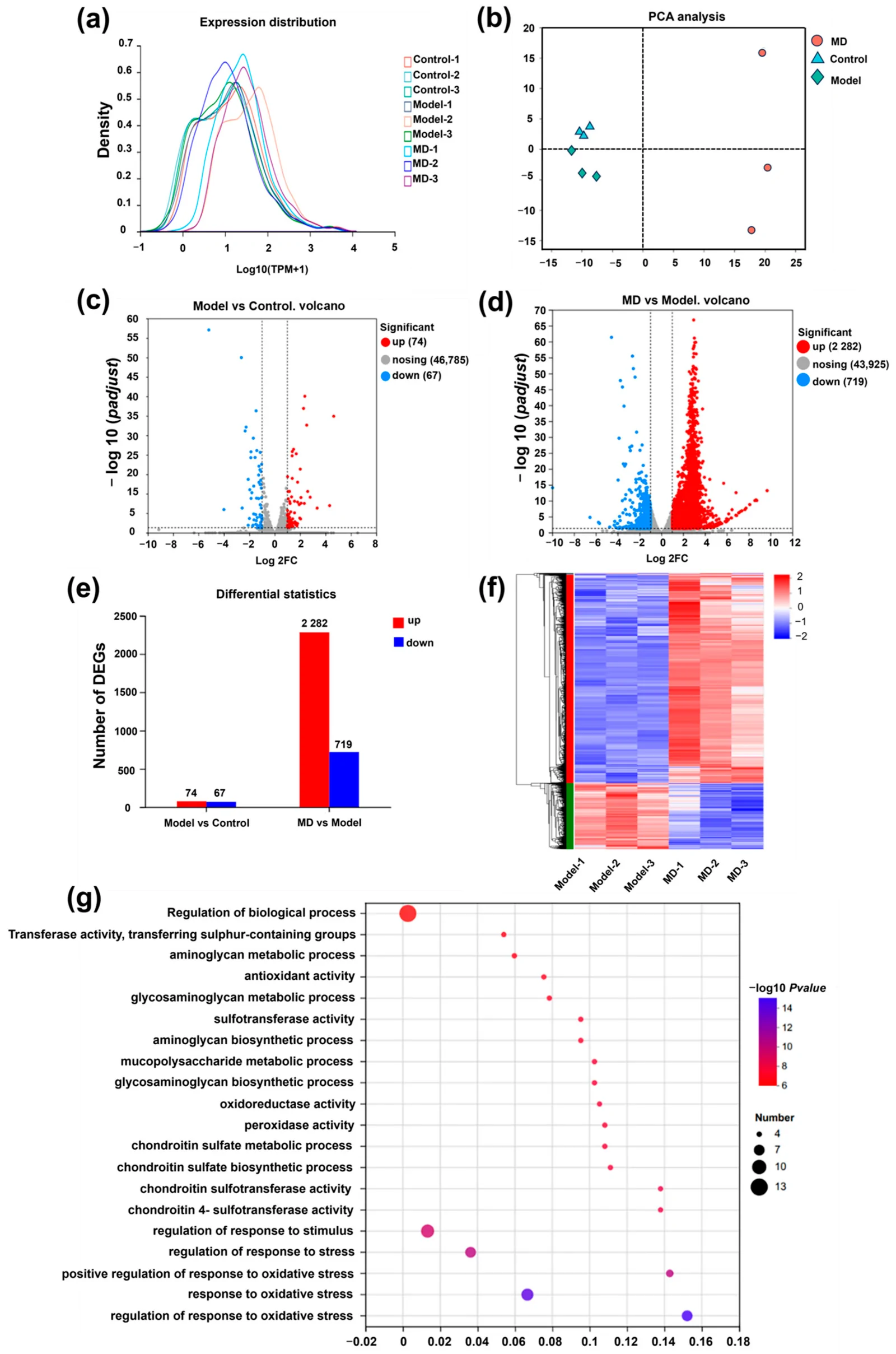
Transcriptomic analysis of *C. elegans* under oxidative stress and polyphenol treatment. (**a**) Density distribution of gene expression levels across all samples. The *x*-axis represents Log10(TPM + 1), where TPM indicates Transcripts Per Million. (**b**) Principal component analysis (PCA) score plot of the Control, Model, and MD groups. (**c**,**d**) Volcano plots of differentially expressed genes (DEGs) for Model vs. Control (**c**) and MD vs. Model (**d**). The *x*-axis represents log2 fold change (log2FC), and the *y*-axis represents the negative log10 of the adjusted *p*-value (−log10 padjust). Red, blue, and gray dots indicate significantly upregulated, downregulated, and non-significant (nosig) genes, respectively. (**e**) Statistical histogram showing the number of upregulated (red) and downregulated (blue) DEGs in the two comparison groups. (**f**) Hierarchical clustering heatmap of DEGs between the MD and Model groups. Rows represent genes, and columns represent biological replicates. The color gradient indicates the scaled expression levels. (**g**) Gene ontology (GO) enrichment bubble chart of DEGs. The *x*-axis represents the enrichment factor, the bubble size indicates the number of genes, and the color gradient represents the significance level (−log10 *p*-value).

Based on the above screening results, this study further performed transcriptome sequencing on the oxidative stress model group, the polyphenol intervention (MD) group, and the blank control group to explore the molecular mechanisms. Transcriptome sequencing data quality control showed that the A_260_/A_280_ of RNA in all samples were ≥1.8, the sequencing quality values Q20 > 98%, Q30 > 95%, and the base distribution was balanced, indicating that the RNA had high purity and good quality, which could be used for subsequent analysis. The overall outline of the TPM density distribution curve of each sample was similar (Figure 5a). Principal component analysis (PCA, Figure 5b) showed that the samples of oxidative stress model group, polyphenol intervention (MD) group and blank control group gathered and separated from each other, indicating that the gene spectrum of nematodes changed significantly under oxidative stress. The volcano plot also confirms this point (Figure 5c,d).

Differential gene analysis showed that only 141 differentially expressed genes (74 up-regulated and 67 down-regulated) were detected in the model group compared with the blank group. Compared with the model group, there were 3001 differential genes in the MD group, of which 2282 were up-regulated and 719 were down-regulated (Figure 5e), suggesting that polyphenol intervention triggered a wide cascade response at the transcriptional level. After functional enrichment of the differentially expressed genes in the MD group (Table 2), they were significantly mapped to biological processes such as carbohydrate metabolism, amino acid metabolism, protein folding, classification and degradation, translation, and signal transduction, which is consistent with the general understanding of polyphenol regulation metabolism and stress response. Focusing on stress-related genes, we locked multiple candidate molecules such as skpo-3, ZK1025.8, tsp-17, F49D11.6, djr-1.2, eya-1, ets-7, pxn-1, C46A5.4 and utx-1 from the differential genes. The following discussion focuses on eya-1, utx-1, cyp-35A2 and tsp-17, which are closely related to oxidative stress.

EYA is a highly conserved transcriptional cofactor and protein phosphatase that regulates multiple developmental processes throughout metazoa. The protein encoded by eya-1 has homology with mammalian EYA1-4 [29]. In this study, eya-1 in MD group was significantly down-regulated, suggesting that polyphenols may be involved in the regulation of apoptosis and stress response. Wang et al. (2014) found that knocking down eya-1 could lead to a significant decrease in the tolerance of nematodes to high temperature and oxidative stress, and under oxidative stress conditions, knocking down eya-1 could shorten the average lifespan by 18.7% [30]. This study confirmed that eya-1 is an essential factor for nematode resistance, which may function down-stream of daf-16 and regulate a series of stress-resistant genes. It is therefore speculated that eya-1 may be a potential target through which polyphenols affect oxidative stress resistance in *C. elegans*; however, this remains a hypothesis and requires further vali-dation.

Utx-1 belongs to the conserved histone demethylase family and specifically catalyzes the demethylation of H3K27me3, which is a marker associated with chromatin inhibition [31]. The expression of utx-1 in MD group was significantly down-regulated, suggesting that the apoptotic signal caused by oxidative stress may be weakened. Jin et al. (2011) confirmed that knockout or heterozygous mutation of utx-1 could prolong the lifespan of *C. elegans* and increase the overall level of H3K27me3, and established it as a negative regulator of somatic cell lifespan [32]. Maures et al. (2011) further revealed that utx-1 regulates lifespan by removing the H3K27me3 inhibitory marker on the key gene daf-2 of the insulin/IGF-1 signaling pathway (IIS) [33]. Taken together with the finding that polyphenols extend the lifespan of *C. elegans* under oxidative stress, we speculate that utx-1 may be one of the potential genes associated with the effects of polyphenols on lifespan.

*cyp-35A2* belongs to the CYP35 cytochrome P450 subfamily and is highly homologous to the human CYP2 family. It is widely involved in fatty acid metabolism, xenobiotic detoxification and oxidative stress response [34]. In the MD group, *cyp-35A2* was significantly upregulated, suggesting that polyphenol treatment may enhance the organism’s active defense against oxidative stress, increase oxidoreductase activity, and help reduce oxidative damage. This result is consistent with the upregulation of detoxification metabolic pathways observed in transcriptomic studies of other polyphenols [35], and similar detoxification metabolic pathways were also observed in nematode transcriptome studies of other polyphenols (such as quercetin and resveratrol).

*Tsp-17* encodes a member of Tetraspanin four transmembrane protein family, which is involved in cell migration, adhesion, intracellular transport and neuroprotection. The gene was significantly down-regulated in the MD group and participated in the negative regulation of oxidative stress. Studies have shown that *glit-1* and *tsp-17* are in the same genetic pathway [36]. Both *glit-1* and *tsp-17* mutants exhibit a non-additive hypersensitivity phenotype to 6-OHDA and paraquat-induced oxidative damage, and the oxidative stress reporter gene is strongly activated. The down-regulation of *tsp-17* may be involved in the regulation of oxidative damage in nematodes in a feedback manner, and its specific contribution in polyphenol-mediated protective effects still needs to be further explored.

In general, polyphenol intervention profoundly changed the transcriptional network of *C. elegans* under oxidative stress. It not only up-regulated the detoxification defense system represented by *cyp-35A2*, but also down-regulated *eya-1*, *utx-1* and *tsp-17*, which may be related to apoptosis regulation, epigenetic modification and stress signal transduction. Combining these transcriptome clues with the longevity phenotype provides a clear direction for the next step to target the functional targets of polyphenols to alleviate oxidative stress.

In order to systematically grasp the functional trend of the transcriptome after polyphenol intervention, GO functional annotation was performed on the differential genes between the MD group and the model group. Among the three ontologies of biological process, molecular function and cellular component, the entries related to oxidative stress were highly enriched, and the number of differentially expressed genes in biological process was the largest and the significance was the strongest. Among them, 145 biological processes were up-regulated. These processes involve ‘regulation of biological process’ (GO: 1902882), ‘response to oxidative stress’ (GO: 0006979), ‘regulation of biological process’ (GO: 0050789) and ‘regulation of response to stimulus’ (GO: 0048583). Cellular component and molecular function analyses further identified a large number of genes annotated with “antioxidant activity,” “peroxidase activity,” and “transferase activity,” suggesting that the transcriptional response to polyphenols is accompanied by changes in the redox defense network.

**Figure 6:**
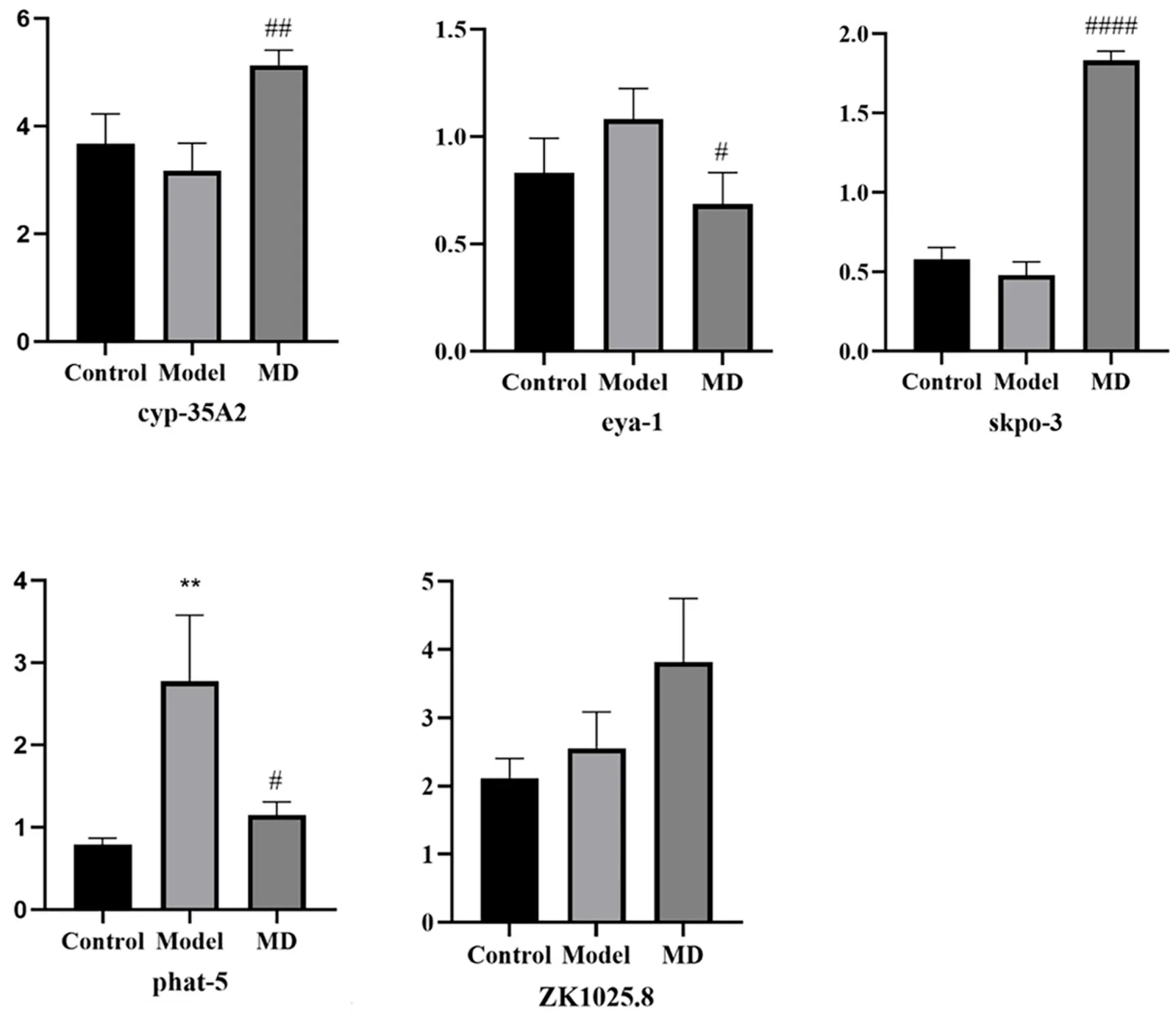
Effects of purified millet polyphenol extract on the relative expression levels of antioxidant-related genes in *Caenorhabditis elegans*. The vertical axis represents the relative expression levels of the respective genes (*cyp-35A2*, *cya-1*, *skpo-3*, *phat-5*, and *ZK1025.8*). Data are presented as mean ± standard deviation. Compared with the Control group, ** *p* < 0.01; compared with the Model group, *# p* < 0.05, ## *p* < 0.01, and #### *p* < 0.0001 (using one-way ANOVA followed by post hoc tests).

In order to verify the experimental results of RNA-Seq, five genes related to stress response were selected from differentially expressed genes for verification. As shown in Figure 6, compared with the model group, the mRNA levels of *cyp-35A2*, *skpo-3* and *ZK1025.8* in nematodes were significantly increased after polyphenol treatment, while the expression of *eya-1* and *phat-5* was significantly decreased. The change trend of each gene was completely consistent with RNA-Seq data, confirming the reliability of transcriptome sequencing in this study.

KEGG pathway analysis further revealed that the antioxidant effect of polyphenols was not dependent on a single signal cascade, but simultaneously mapped to multiple pathways such as Wnt, PI3K-Akt, P53/P21, cGMP-PKG, Hippo, mTOR and FoxO. Among them, Wnt signaling pathway plays a central role in the regulation of cell proliferation, differentiation and apoptosis [37]. In the KEGG map of this study, the expression of glycogen synthase *kinase-3β* (GSK-3β) was up-regulated after polyphenol treatment. GSK-3β is a serine/threonine protein kinase involved in protein synthesis, glycogen metabolism, mitosis and apoptosis [38]. The activation of GSK-3β promotes the assembly of β-catenin degradation complexes, thereby inhibiting the excessive activation of Wnt/β-catenin signaling. It has been reported that polydatin also alleviates oxidative damage in mouse spinal cord injury by activating the Wnt/β-catenin pathway [39]. Therefore, the antioxidant effects of polyphenols may be associated with changes in the Wnt signaling pathway, but the specific functional contribution of this pathway remains to be experimentally confirmed.

The PI3K-Akt pathway is also an important hub for regulating cell proliferation, metabolism and apoptosis. In the pathway model constructed in this study, the upstream IRS protein can enhance PI3K activity, convert phosphatidylinositol diphosphate (PIP2) into phosphatidylinositol 3,4,5-triphosphate (PIP3), and then recruit and activate Akt. On the one hand, activated Akt can phosphorylate Caspase-9 and inactivate it, thereby blocking the apoptosis cascade [40]; on the other hand, the anti-apoptotic effect can be further enhanced by inhibiting GSK-3 activity [41]. Studies have shown that activation of the PI3K/Akt pathway can reduce oxidative stress-induced cell damage. For example, allicin reduces H_2_O_2_-induced oxidative stress in osteoblasts and maintains mitochondrial function through this pathway [42]. These findings suggest that the antioxidant effects of polyphenols may be associated with changes in the PI3K-Akt pathway, but whether apoptosis is inhibited through this pathway still requires functional experimental validation.

In addition, the differential genes in this study were significantly enriched in the P53/P21 pathway. P53 is activated under DNA damage, excessive proliferation or inflammatory stimulation, and transcriptionally up-regulates P21, which binds to the Cyclin-CDK complex and inhibits the activity of CyclinD1-CDK4/6 and CyclinE-CDK2, leading to cell cycle arrest [43]. Accordingly, it is speculated that the antioxidant effects of polyphenols may be associated with changes in the p53/p21 signaling pathway.

KEGG analysis also enriched cGMP-PKG, Hippo, mTOR and FoxO signaling pathways, among which FoxO pathway was most directly related to oxidative stress and lifespan regulation in *C. elegans*. FoxO transcription factors are highly conserved in evolution and are involved in multiple processes such as cell cycle arrest, apoptosis, and anti-oxidative stress [44]. In *C. elegans*, FoxO homolog DAF-16 is a key effector downstream of the insulin/IGF-1 signaling pathway. In this study, we found that eya-1 functions downstream of DAF-16, while utx-1 affects the activity of insulin/IGF-1 signaling by removing H3K27me3 modification [32]. Both genes were significantly changed after polyphenol treatment. At the same time, the mTOR pathway also appears in the enrichment list, and its regulation of autophagy and protein synthesis is closely related to lifespan. These results suggest that the effects of polyphenols may not depend on a single pathway, but may be associated with changes in multiple signaling networks, such as FoxO and mTOR, thereby linking oxidative stress responses and lifespan regulation in *C. elegans* at multiple levels.

In summary, through transcriptome sequencing and experimental verification, this study confirmed that polyphenols can simultaneously regulate key nodes such as cyp-35A2, eya-1, utx-1, and tsp-17, and systematically activate multiple signaling pathways such as Wnt, PI3K-Akt, P53/P21, and FoxO, thereby enhancing the defense ability of nematodes against oxidative stress. This multi-target and multi-pathway synergistic mode of action provides a rich transcriptome basis for analyzing the antioxidant and longevity mechanism of polyphenols, and also points out the direction for the accurate verification of subsequent functional genes.

### 3.8. Effect of Millet Polyphenols on Metabolism of Caenorhabditis elegans

**Figure 7:**
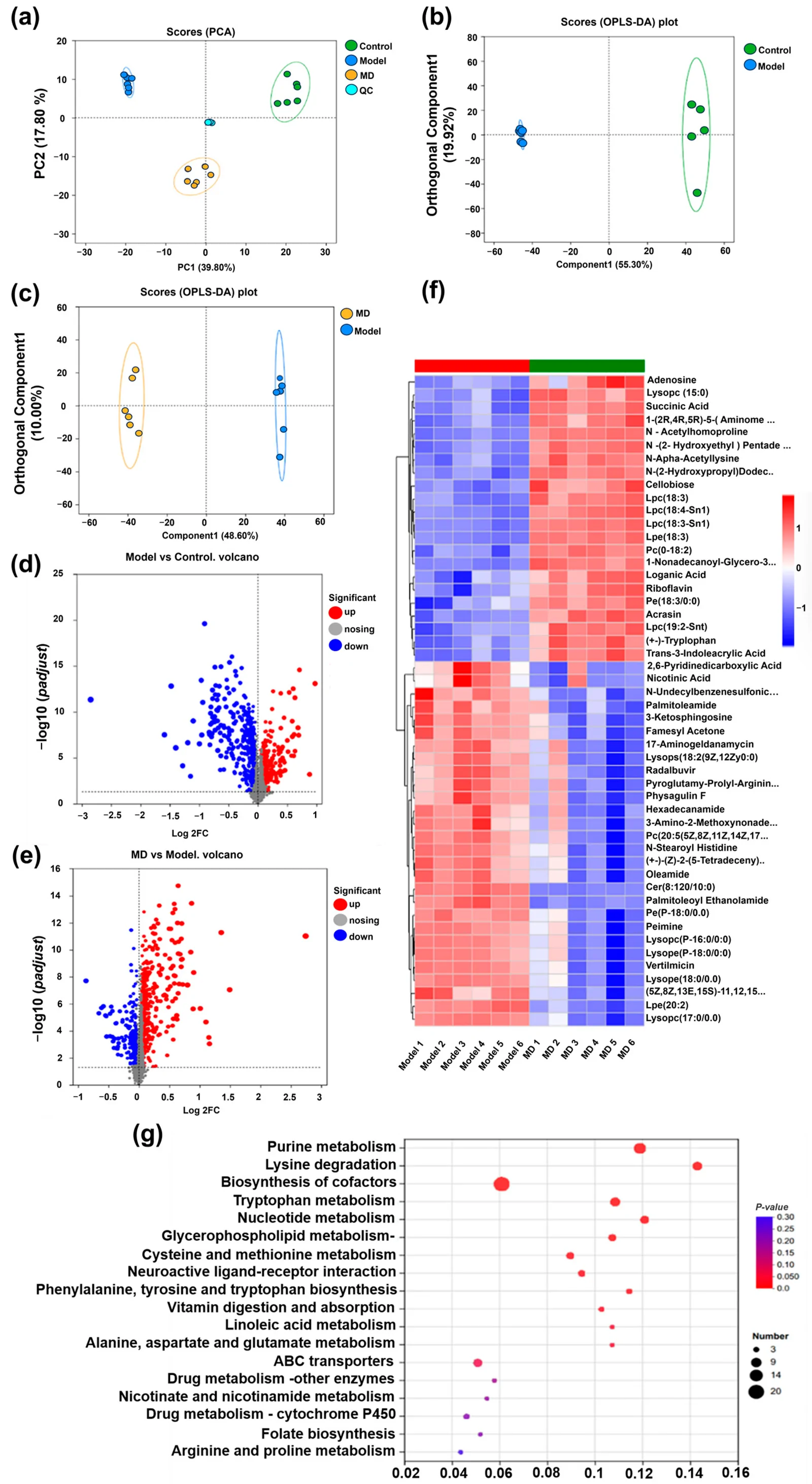
Non-targeted metabolomics analysis of *C. elegans*. (**a**) PCA score plot across the Control, Model, MD, and Quality Control (QC) groups. (**b**,**c**) OPLS-DA score plots for Model vs. Control (**b**) and MD vs. Model (**c**). (**d**,**e**) Volcano plots for Model vs. Control (**d**) and MD vs. Model (**e**). The *x*-axis represents log2 fold change (log2FC), and the *y*-axis represents the negative log10 of the Benjamini–Hochberg (BH)-adjusted *q*-value. Red, blue, and gray dots indicate significantly upregulated, downregulated, and non-significant (nosig) metabolites, respectively (VIP > 1, BH-adjusted *q* < 0.05). (**f**) Heatmap of the top 50 differential metabolites between the MD and Model groups. (**g**) Kyoto Encyclopedia of Genes and Genomes (KEGG) pathway enrichment bubble chart (Top 20). The bubble size indicates the number of metabolites, and the color gradient indicates the *p*-value. (Note: All metabolites are annotated at MSI Level 2 as putative identifications).

After 7 cycles of cross-validation of metabolomics data, the quality control samples were closely clustered in the PCA score plot (Figure 7a), and the correlation was good, indicating that the instrument had high stability during the detection process. Under the same PCA model, there was a significant separation trend between the blank group, the model group and the MD group, indicating that the metabolite profile of nematodes changed significantly under different treatment conditions.

In order to focus on the differences between groups, orthogonal partial least squares discriminant analysis (OPLS-DA) was further used to model. As shown in Figure 7b, the metabolic spectrum of *C. elegans* changed after H_2_O_2_ treatment, indicating that the metabolic spectrum of *C. elegans* under normal conditions could be significantly changed under H_2_O_2_ stress. Figure 7c showed that the metabolic profile of nematodes changed after polyphenol treatment, indicating that polyphenol treatment changed the metabolic profile of nematodes under oxidative stress. The Q2 of the two models is greater than 0.5, and the explanatory power and predictive ability of the model meet the requirements of experimental analysis.

The overall distribution of candidate differential metabolites can be visually seen from the volcano plot (Figure 7d,e). Compared with the normal group, a total of 744 candidate differential metabolites (393 up-regulated and 351 down-regulated) were identified in the model group. Compared with the model group, there were 716 candidate differential metabolites (491 up-regulated and 225 down-regulated) in the MD group. The number of candidate differential metabolites in the two groups was basically in the same order of magnitude, but the proportion of up-regulated metabolites in the MD group was higher, suggesting that polyphenol intervention may actively induce the activation of some protective metabolic pathways.

Hierarchical cluster analysis was performed on the top 50 candidate differential metabolites with VIP values (Figure 7f). The results showed that these substances were mainly concentrated in carboxylic acids and their derivatives, purine nucleosides, indoles and derivatives, glycerophospholipids and other categories.

Guided by the transcriptome results, we used VIP > 1 and BH-adjusted q < 0.05 as screening criteria and identified a set of key candidate differential metabolites between the MD group and the model group. Because no authentic standard confirmation was performed in this study, the identities of these metabolites are all MSI Level 2 putative identifications. These metabolites were mainly distributed in pathways such as glycerophospholipid metabolism, purine metabolism, ABC transporters, and tryptophan metabolism, providing potential associative clues for the antioxidant effects of polyphenols at the metabolic level. The interpretations of these pathways are presented here as hypotheses.

A total of 15 candidate differential metabolites were detected in the glycerophospholipid metabolic pathway in the MD group. Glycerophospholipids are not only the structural basis of biofilms, but their metabolic remodeling is also closely related to energy supply and oxidative stress adaptation. Cheng et al. (2016) found that the synthesis of glycerophospholipids such as PC and PE in tumor cells is often up-regulated to meet the material and energy needs of rapid proliferation [45]; li et al. (2023) confirmed that nucleotide yeast hydrolysates can prolong the lifespan of nematodes and enhance antioxidant capacity by regulating glycerophospholipid metabolism [46]. The widespread changes in glycerophospholipid metabolites after polyphenol intervention suggest that polyphenols may be involved in actively adjusting membrane lipid composition or signaling lipid metabolism, thereby potentially helping cells maintain homeostasis under oxidative stress.

Purine metabolism also changes significantly. In the MD group, the contents of 13 metabolites such as adenosine, uric acid, flavin and adenine were significantly changed. Once the recovery of purine is out of balance with de novo synthesis, it will directly aggravate the production of ROS [47]. As the end product of purine metabolism, uric acid has strong free radical scavenging ability at physiological concentration [48]. Recent studies have also shown that *Cyclocodon lancifolius* extract exerts the anti-aging activity of nematodes by regulating purine metabolism [49]. The overall upregulation of purine metabolites after polyphenol treatment suggests that the organism may enhance the salvage pathway or uric acid synthesis, thereby increasing its endogenous antioxidant buffering capacity; however, this still requires validation by functional experiments.

It is worth noting that the up-regulated metabolites of adenosine, cellobiose, riboflavin, flavin, maltotriose and thiamine in the MD group belong to the substrates or related molecules of ABC transporters. The ABC transporter family is responsible for the transmembrane transport of a variety of endogenous and exogenous substances, plays an important role in membrane lipid transport, oxidative stress defense and detoxification [50], and can remove toxic compounds by effluxing GSH conjugates [51]. The synergistic up-regulation of these substrates suggests that polyphenols may strengthen the ABC transporter-mediated detoxification and antioxidant protection network.

Multiple nodes of tryptophan metabolism are also regulated by polyphenols. The contents of 5-methoxytryptamine and L-tryptophan in MD group increased, while indole-3-acetamide decreased. Tryptophan metabolism is widely involved in protein synthesis, inflammation regulation and oxidative stress response [52], and its derived indoles are important molecules for maintaining intestinal barrier and antioxidant defense [53]. Polyphenol modulation of tryptophan metabolism suggests a possible role in inhibiting inflammation and reducing oxidative damage, but the causal mechanisms remain unclear.

In addition, changes in metabolites such as succinic acid, quinolinic acid and biliverdin were also detected in the MD group. Succinic acid is not only an intermediate product of the tricarboxylic acid cycle, but also participates in the regulation of oxidative stress and inflammation as a signaling molecule [54]. Bilirubin and its reducing product bilirubin are important endogenous antioxidants, which can directly scavenge a variety of ROS, reduce cisplatin-induced apoptosis in renal tubular cells [55], and alleviate inflammation by inhibiting IL-18 and macrophage M1 polarization [56]. The changes in these metabolites suggest that polyphenols may jointly affect the redox balance of *C. elegans* through two dimensions: energy metabolism and antioxidant defense. These pathways are regarded as candidate mechanisms and still require functional validation.

The results of pathway enrichment and topological analysis of candidate differential metabolites are shown in Figure 7g, which lists the top 20 pathways most affected. Among them, those strongly associated with oxidative stress mainly include cofactor biosynthesis, purine metabolism, glycerophospholipid metabolism, amino acid biosynthesis, tryptophan metabolism, nucleotide metabolism, ABC transport and drug metabolism-cytochrome P450. These metabolic changes are consistent with the response trends of Wnt, PI3K-Akt, FoxO, and other signaling pathways observed in the transcriptome, jointly suggesting that polyphenols may be associated with the regulation of oxidative stress in *C. elegans* at both the transcriptional and metabolic levels. However, this remains an association analysis at the transcriptomic and metabolic levels, and the exact causal mechanisms still need to be further explored through experiments such as gene knockdown or mutant studies.

## 4. Discussion

The antioxidant mechanisms of cereal polyphenols have been widely reported, but different cereals may differ in their main modes of action. For example, some studies on purple wheat have reported that the effects of its polyphenol components are often related to the regulation of transcription factors such as DAF-16/FOXO [57], whereas anthocyanins from black rice have been shown to exert antioxidant and lifespan-extending effects through the insulin/IGF-1 signaling (IIS) pathway [58]. Unlike previous findings that mostly focused on a single pathway, this study found that purified foxtail millet polyphenols not only improved the physiological indicators of *C. elegans*, but their transcriptomic data also suggested changes in multiple pathways, including Wnt, PI3K-Akt, and p53/p21. At the same time, the fluctuations in glycerophospholipid and purine metabolism in the metabolomic results are also consistent with previous reports that polyphenols from fermented red rice and fermented barley affect lipid and energy metabolism [59,60]. These results suggest that the antioxidant effects of foxtail millet polyphenols are related not only to the basic regulatory networks shared by cereal polyphenols (such as the IIS pathway), but are also accompanied by broader transcriptional and metabolic responses.

This multi-pathway regulation and metabolic remodeling, together with the green extraction process used in this study, suggest that foxtail millet polyphenols may have potential as high-value natural ingredients, providing preliminary scientific clues for the development of functional foods aimed at improving redox balance and metabolic homeostasis.

First, this study did not include a separate NADES blank control or controls for individual NADES components; therefore, the observed effects cannot be fully attributed to polyphenol monomers alone. Second, only a single MD concentration with the best overall phenotype was used for omics analysis. Although this can provide reliable molecular evidence under this condition, it cannot fully characterize dose-dependent effects, and the related molecular features should not be directly extrapolated. Third, the key metabolites were not verified with authentic standards (they belong to MSI Level 2 putative identifications). Most importantly, no functional experiments such as gene knockdown, mutant strains, or pathway inhibitors were performed. Therefore, the enriched pathways, including Wnt, PI3K-Akt, p53/p21, and FoxO, are only suggestive candidate mechanisms, and their activation status or causal involvement cannot yet be established. Future work will incorporate proper NADES blank and component controls, and will use RNAi knockdown or mutant experiments targeting key pathways, combined with biochemical validation, to further clarify the direct molecular targets of polyphenols, the specific contribution of key genes to antioxidant phenotypes, and the causal relationship between metabolite changes and physiological outcomes.

## Figures and Tables

**Table 1:** Deep eutectic solvent preparation table.

	Component 1	Component 2	Component 3	Molar Ratio
NADES-1	Choline chloride	Glucose	Fructose	1:1:1
NADES-2	Glycerol	Malic acid	Glucose	1:1:1
NADES-3	Glycerol	Citric acid	Glucose	1:1:1
NADES-4	Citric acid	Fructose	Glucose	1:1:1
NADES-5	Citric acid	Malic acid	Fructose	1:1:1
NADES-6	Malic acid	Fructose	Glucose	1:1:1
NADES-7	Malic acid	Citric acid	Glucose	1:1:1
NADES-8	Glycerol	Citric acid	Fructose	1:1:1
NADES-9	Glycerol	Malic acid	Fructose	1:1:1

NADES stands for Natural Deep Eutectic Solvents, with the original abbreviation retained in Table 1.

**Table 2:** List of DEGs associated with antioxidant activity in the MD.

ID	Name	RNA-Seq FC	Adjusted *p* Value
WBGene00007813	*jmjd-3.3*	2.69	0.002
WBGene00043302	*F32D8.11*	3.18	<0.001
WBGene00006555	*tbx-36*	0.49	<0.001
WBGene00006643	*tsp-17*	0.46	0.015
WBGene00010606	*cyp-13B2*	0.48	0.003
WBGene00020242	*phat-5*	0.45	<0.001
WBGene00014188	*ZK1025.8*	2.02	<0.001
WBGene00016189	*sprp-4*	0.36	0.001
WBGene00016789	*djr-1.2*	3.81	0.015
WBGene00001377	*eya-1*	0.43	<0.001
WBGene00017601	*ets-7*	0.18	0.001
WBGene00009110	*F25D1.5*	0.27	0.002
WBGene00007929	*dmd-10*	2.62	0.008
WBGene00000449	*ceh-27*	0.38	<0.001
WBGene00001210	*egl-46*	0.45	<0.001
WBGene00018630	*F49D11.6*	2.12	0.001
WBGene00010166	*gmd-2*	0.26	<0.001
WBGene00009271	*glna-3*	3.19	<0.001
WBGene00012723	*Y39G8B.2*	0.47	<0.001
WBGene00019489	*K07E1.1*	2.03	<0.001

DEGs were identified using the criteria of |log2FC| ≥ 1 and Benjamini–Hochberg (BH)-adjusted *p* value < 0.05. FDR, false discovery rate.

## Data Availability

The original contributions presented in this study are included in the article. Further inquiries can be directed to the corresponding author.

## Abbreviations

The following abbreviations are used in this manuscript:

NADES	natural deep eutectic solvent
*C. elegans*	*Caenorhabditis elegans*
